# The moral self and moral duties

**DOI:** 10.1080/09515089.2020.1789577

**Published:** 2020-07-13

**Authors:** Jim A. C. Everett, Joshua August Skorburg, Julian Savulescu

**Affiliations:** a Department of Social and Organisational Psychology, Leiden University; b School of Psychology, University of Kent; c Department of Philosophy, Duke University; d Department of Philosophy, Guelph University

**Keywords:** Personal identity, morality, moral self, experimental philosophy

## Abstract

Recent research has begun treating the perennial philosophical question, “what makes a person the same over time?” as an empirical question. A long tradition in philosophy holds that psychological continuity and connectedness of memories are at the heart of personal identity. More recent experimental work, however, has suggested that persistence of moral character, more than memories, is perceived as essential for personal identity. While there is a growing body of evidence supporting these findings, a recent critique suggests that this research program conflates personal identity with mere similarity. To address this criticism, we explore how loss of someone’s morality or memories influences perceptions of identity change and perceptions of moral duties toward the target of the change. We present participants with a classic “body switch” thought experiment and after assessing perceptions of identity persistence, we present a moral dilemma, asking participants to imagine that one of the patients must die (Study 1) or be left alone in a care home for the rest of their life (Study 2). Our results highlight the importance of the continuity of moral character, suggesting that lay intuitions are tracking (something like) personal identity, not just mere similarity.

## 1. Introduction

The idea of body-switches – waking up with your mind in someone else’s body – has been an enduring feature of popular culture, from children’s books like Mark Twain’s *The Prince and The Pauper* to cult-classic movies like *Freaky Friday*. These stories raise deep questions about what makes a person the same person over time. There is, of course, a long tradition of philosophical debate about these questions. A prominent view, associated with John Locke, posits that *psychological continuity*, specifically, memories, are at the heart of personal identity: Person X at Time 1 can be identified as Person Y at Time 2, if X and Y share an autobiographical memory (Locke, 1975). Indeed, some empirical evidence using body-switch thought experiments indicates that this philosophical perspective tracks folk intuitions, with participants judging that after transplanting a patient’s (Jim) brain into the body of another patient, the patient was “still Jim” when the memories were preserved, but not when memories were erased (Nichols & Bruno, 2010).

More recent work has suggested that while memories are surely important in this regard, so too are other features of the mind. More specifically, moral traits have been proposed as especially identity-conferring (Everett et al., Under Review, Forthcoming; Heiphetz et al., 2018, 2017; Molouki & Bartels, 2017; Matthew et al., 2020; Strohminger et al., 2017; Strohminger & Nichols, 2014, 2015). For example, Strohminger and Nichols (2014) presented participants with a wide variety of traits and asked them to imagine how much a change to a specific trait would influence whether someone is still the same person (e.g., “Jim can no longer remember anything that happened before the accident. Aside from this, he thinks and acts the same way as before the accident. Is Jim still the same person as before the accident?”).

Across five experiments, participants reported that when a person changes in terms of traits like honesty, empathy, or virtuousness, they were rated as more of a different person than when they changed in terms of memories, preferences, or desires. Thus, “moral traits are considered more important to personal identity than any other part of the mind” (Strohminger & Nichols, 2014, p. 168). This “moral self effect” has been demonstrated with 8- to 10-year-olds (Heiphetz et al., 2018), and it holds whether participants are thinking of the self, a friend, or a stranger (Everett et al., Under Review; Heiphetz et al., 2017). This moral self effect is thought to occur in large part because morality is one of the fundamental dimensions on which we perceive others, and changes to morality will likely have much greater influence on the broader community than changes to memories (Everett et al., Under Review; Heiphetz et al., 2017). In short, losing one’s memories is bad for oneself and bad for close friends and family, but losing one’s moral conscience will likely have a much greater negative impact on a much broader range of people.

A recent critique by Starmans and Bloom (2018), however, suggests that this research on the moral self effect rests on a mistake. They argue that the studies fail to distinguish *similarity* and qualitative (or biographical) identity on the one hand, from *personal identity* and quantitative (or numerical) identity on the other (Parfit, 1984). Twins, for example, can be qualitatively identical, but they are still quantitatively distinct people: you might not be able to tell them apart, but they still need two passports to travel abroad. This distinction between numerical and qualitative identity has previously been used to criticize the early studies on the importance of memories to identity persistence (e.g., Blok et al., 2005; Nichols & Bruno, 2010). Berniūnas and Dranseika (2016) argue that these designs do not adequately distinguish between qualitative and numerical identity judgments. For example, when Nichols and Bruno (2010) ask participants “What is required for some person in the future to be the same person as you?,” it is possible that participants are interpreting this question in a qualitative sense, not a numerical one. To test this, they draw on a convenient pair of Lithuanian phrases that disambiguate the two: *tas pats* and *toks pats*. When contrasted with *toks pats, tas pats* means “the same” in the sense of numerical identity, while *toks pats* means “the same” in the sense of qualitative identity. When explicitly disambiguating these two senses to their Lithuanian participants, they found that participants were significantly more likely to agree that someone was the “same person” after losing their memories, suggesting that “retention of memory may not be so critical to the preservation of individual numerical identity” (Berniūnas & Dranseika, 2016, p. 115).

Focusing on the moral self effect research, Starmans and Bloom (2018) draw on the same distinction between numerical identity and similarity to make a more forceful critique, suggesting that previous work has intended to focus on the former, but really has just looked at the latter. If Jim lost his moral conscience after an accident, then it might make sense to say that he seems like a different person. He’s *qualitatively, or biographically*, dissimilar to who he was before the accident. But it wouldn’t make sense, according to Starmans and Bloom, to suggest that post-accident-Jim is *quantitively* distinct from pre-accident-Jim, such that pre-accident Jim’s debts are now forgiven, or that post-accident Jim must now get a new passport. Jim is still the same person, he’s just different from before. In other words, when participants in the “moral self effect” studies report things like “I completely disagree Jim is the same person as before the accident,” they are referring to similarity (qualitative identity) and not strictly personal or quantitative identity.

Starmans and Bloom suggest that this conceptual confusion can be avoided by using well-established paradigms in cognitive and developmental psychology which study the numerical identity of objects. Setting that suggestion to the side, we think there are theoretical ways to respond to Starmans and Bloom’s challenge, namely by calling into question whether it is desirable – or even possible – to strictly divide similarity from numerical identity. We will return to this in the general discussion. For now, we turn our attention to an empirical response to their challenge.

One way to address the adequacy of Starmans and Bloom’s critique on empirical grounds is to look at the *practical consequences* of judgments of identity change. In our recent work, we found that, compared to memories, social changes generally and moral changes specifically not only affected perceptions of identity persistence (as in previous studies), but, crucially, such changes also led participants to subsequently infer a range of practical consequences, including changes in behavior, evaluations by third parties, and reductions in relationship quality (Everett et al., Under Review).

There are many ways in which judgments about identity persistence have practical consequences, as Tobia (2015, 2016)

2016)) and many others have discussed. After all, questions about personal identity are deeply linked with ethical considerations (see, e.g., Shoemaker, 2016). Most obviously, personal responsibility presupposes personal identity: I can only be held morally responsible for *my* actions, not someone else’s. Similarly, consider moral duties to one’s parents. These can be described as special obligations, owed to one’s parents simply because of the relationship one has to them and the persons they are (Jeske, 2014). These special obligations can be distinguished from natural duties, or those “moral requirements which apply to all men [and persons] irrespective of status or of acts performed … owed by all persons to all others” (Simmons, 1979, p. 13). I have obligations to my mother that I don’t have to a stranger, and these special obligations toward my mother don’t change after she colors her hair, changes her job, or undergoes a sex reassignment procedure. My obligations and duties to her are the same because she herself *is* the same person.

Focusing on moral duties in this way may not settle the qualitative versus quantitative debate about personal identity once and for all, but it can circumvent Starman and Bloom’s criticism. If participants are thinking, at least in part, about forensic identity in some Lockean sense (see also discussion) and not just similarity, we should expect that judgments about moral duties should partially track judgments of change. If, for example, I judge that my mother is still the same person after losing her memories, I should also judge that I have the same special obligations toward her. If I judge that she is a different person, in contrast, it seems plausible that my perceptions of special obligation toward her would also change accordingly. In contrast, if Starmans and Bloom are correct that participants are just thinking about similarity and not identity, then participants should rate someone who loses their morals as different, but they should not perceive any changes to their own moral duties toward that person.

## 2. The present studies

Here, we explore how changes to moral traits and memories influence both perceptions of identity change and practical consequences that follow from such judgments. More specifically, we examine perceptions of *moral duties* toward the target of the change. We present participants with a classic “body switch” thought experiment in which a loved one undergoes a brain transplant with a stranger and, as a consequence, either experiences no psychological change, loses all their memories, or loses their moral conscience. After assessing perceptions of identity persistence, we present a moral dilemma, asking participants to imagine that one of the patients must die (Study 1) or be left alone in a care home for the rest of their life (Study 2). Participants must decide who to save or care for: the patient with their partner’s brain and the stranger’s body, or the patient with the stranger’s brain and the patient’s body.

## 3. Study 1

### 3.1. Method

#### 3.1.1. Open science

All measures, manipulations, and exclusions, and all data, analysis code, supplementary results, and experiment materials are available for download at: https://osf.io/wehkn/

#### 3.1.2. Design

296 American participants completed the survey online using Amazon Mechanical Turk and received 0.80 USD for their time. Participants were excluded from analysis if they took the survey more than once (*N* = 6), leaving a final sample of 290 participants (108 female, *M*_age_ = 33, *SD* = 8.84). This study had three conditions: one where the patient had no discernable change after the operation (“control”), one where the patient lost all of their memories (“memories”), and one where the patient lost their moral conscience (“morality”). At the start of the study, participants were told that for this task, we needed them to think of someone that is very close to them. Participants were told that it did not matter who the person they’re thinking of is or what their specific relationship to the participant is, as long as they are close to the participant. Participants were given a list of 14 options of the person they were thinking of, which we hoped would cover most, if not all, the people participants could think of (mother; father; son; daughter; brother; sister; husband; wife; partner; friend; grandmother; grandfather; cousin; aunt; uncle).

After selecting and confirming their decision, participants were told that they would be taking part in a short imagination exercise. The story revolved around a “mad scientist” who had kidnapped a number of people, including the person that the participant indicated they were thinking about. In the story (and for all the subsequent measures) we used piped text to feed back the person into the story (e.g., “There is a mad scientist who has kidnapped a number of people, including your [friend/husband/aunt, etc.]” This was done to enhance the plausibility of what is, of course, quite an unusual story. For the remainder of the paper we shall refer to the person the participant was thinking of simply as their “partner.” In all three conditions, the story began the same way:

There is a mad scientist who has kidnapped a number of people, including your [partner]. The scientist plans to test a new – and unapproved – procedure that would allow full brain transplants. The scientist knows that if this works it would revolutionise medicine, but because the procedure is risky they have not been able to find willing participants. Because the scientist has not found anyone who is willing, they have kidnapped a group of people, including your [partner] and is going to perform the procedure on them. The scientist performs the operation, and places the brain of your [partner] into a stranger’s body, and places the stranger’s brain into your [partner’s] body. The operation appears successful. All the right neural connections have been made, and after testing all physiological responses the scientist determines that the patients are alive and functioning.

The next part of the story differed by condition. In the control condition, participants were told that there was no psychological change: “The patient with your [partner’s] brain and the stranger’s body thinks and acts just like your [partner] did before the operation.” In the ‘memories’ condition, participants were told that “both patients have completely lost all of their memories: neither patient can remember anything that has happened to them before the operation,” though apart from this they are the same. Finally, in the ‘moral trait’ condition, participants were told that “both patients have completely lost all of their moral consciences: neither patient cares about the people they used to care about, and shows no emotional reaction when the scientist threatens to torture and hurt those people. Both patients appear to no longer be capable of judging right from wrong, or being moved by the suffering of others,” although apart from this they are the same.

After completing questions concerning the psychological persistence of their partner, participants were then introduced to the second part of the story. This part was also identical across the conditions. Participants were told the following:

After the operation on the stranger and your [partner], the scientist’s actions were discovered. Luckily, the other people they kidnapped were not operated on. While being captured, the scientist was killed and the two patients (the stranger and your [partner]) were taken into hospital to be monitored. Unfortunately, while the patients seemed fine at first, doctors discover serious abnormalities. Looking through the things of the scientist, they discover that the scientist had anticipated this and had developed an injection that would cure this. There is, however, only enough for one person, and with the scientist being dead, the doctors cannot create more. One of the patients must die.

After reading this, participants were asked some questions about which patient they should save. Finally, on a new page, participants were told to “assume that the patient with your [partner’s] brain and the stranger’s body is your ‘real’ [partner] and this is the patient you decided to save.” Then, they were asked questions about whether their partner is now the same person as before, whether they will behave differently compared to how they do now, and whether their relationships will change from how they are now.

### 3.2. Measures

#### 3.2.1. Psychological identity persistence

First, participants completed a categorical identification question in which they were asked to identify “which of the two patients is your [partner]?” Participants had three options for this question: the patient with the partner’s brain and the stranger’s body, the patient with the stranger’s brain and the partner’s body, or neither.

Second, we measured psychological identity persistence with three items which measured whether participants thought that the patient who now had the brain of their partner was, in fact, their partner. First, participants were asked “To what extent is this patient with your [partner’s] brain and the stranger’s body your [partner]?^1^” (*1 = definitely not my partner; 7 = definitely is my partner*). Second, participants were asked “To what extent is this patient with your [partner’s] brain and the stranger’s body identical to your [partner]?” (*1 = completely different; 7 = completely identical*). Third, participants were asked “To what extent is this patient with your [partner’s] brain and the stranger’s body the same person they were before?” (*1 = completely different; 7 = completely identical*). Scores on these three questions show the same pattern of results and are positively correlated (all *r*s >.45, *p*s < .001), so we have combined these scores into a reliable composite measure of psychological identity persistence (α = .75). Results with each sub-item individually show the same pattern and are reported in full in the supplementary results.

#### 3.2.2. Moral duties

To measure participants’ sense of moral responsibility toward the patients, participants were first asked two categorical questions: first, which of the patients (if any) they should choose to let live; and second, which of the patients (if any) they had a duty to protect. For both questions, participants had three options: the patient with the partner’s brain and the stranger’s body, the patient with the stranger’s brain and the patient’s body, or neither. Third, participants were asked to indicate how morally wrong it would be to sacrifice the patient with the partner’s brain (*1 = not at all morally wrong; 7 = extremely morally wrong*).

#### 3.2.3. Consequences of identity change

Finally, we had participants assume that they saved the patient with their partner’s brain and the stranger’s body and asked two questions assessing how they expected their partner’s social life and interactions to change as a result of the operation. First, we asked participants to what extent their partner’s relationships will change from how they are now, and second, to what extent their partner will now behave differently (*1 = not at all, 7 = very much)*.

### 3.3. Results

#### 3.3.1. Identity persistence

First, we looked at identity persistence. Across all conditions, participants adopted a psychological account of identity persistence, with most participants judging that the “real” partner was the patient with their partner’s brain and the stranger’s body, and few judging that the “real” partner was the one with the stranger’s brain and their partner’s body (< 9%; see Table 1). Compared to the control condition (18%), participants were more likely to judge that *neither* patient was their real partner (40%) when they had completely lost their moral conscience, *x*^2^(1) = 7.14, *p*= .008. In the ‘moral trait’ condition – but not in the control or ‘memories’ conditions – there was no significant difference between the number of participants who said that the partner-brain patient (i.e., the patient with the partner’s brain and the stranger’s body) was the real partner (51%) and the number of participants who said that neither was the real partner, *x*^2^(1) = 1.45, *p*= .23.

**Table 1.** Percentage of participants in Study 1 judging that each (or neither) patient was the “real” partner, as a function of whether they experienced no psychological change (control condition), lost their memories, or lost their morals.

	*“Which of the two patients is your partner?”*		
	Patient with the partner’s brain and stranger’s body	Patient with the stranger’s brain and partner’s body	Neither
Control	76%	6%	18%
Loss of Memories	63%	5%	32%
Loss of Morals	51%	9%	40%

**Figure 1.**
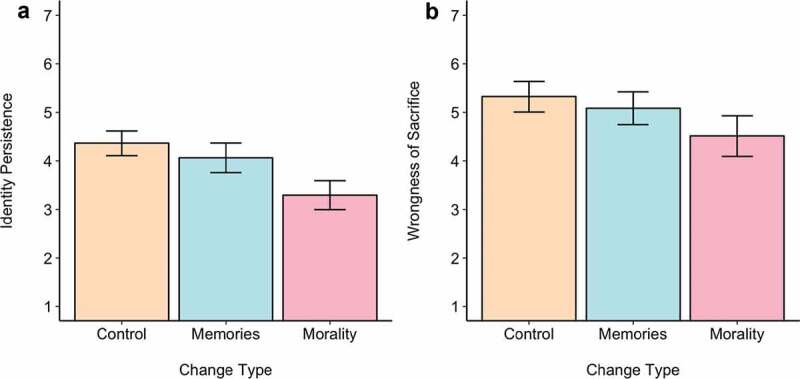
Perceived identity persistence and wrongness of sacrificing as a function of condition (no psychological change; loss of memories; loss of moral conscience). Higher scores indicate more perceived identity persistence (1a) and more perceived moral wrongness (1b). Error bars represent 95% confidence intervals.

Similar results were found when looking at ratings of how much the patient with the partner’s brain and the stranger’s body was, in fact, the real partner (see Figure 1(a)). We observed a significant effect of the ‘change’ condition, *F*(2,287) = 14.90, *p*< .001, η_p_^2^ = .09, whereby participants were significantly more certain that the partner-brain patient was their real partner when they had not changed at all, as compared to when they had changed morally (*t*= 5.31, *p*< .001) or lost their memories (*t*= 3.78, *p*= .008). In contrast, there is no difference in ratings of identity persistence between the control and the ‘memories’ condition (*t*= 1.48, *p*= .30). Together, these results highlight that people perceived that continuity of the mind is more important than continuity of the body, but within this, continuity of one’s moral conscience is perceived to be more important than continuity of one’s memories.

#### 3.3.2. Moral duties

Second, we looked at perceived moral duties toward the patients. When asked who to save, across all conditions, the majority of participants said that if forced to decide, they would save the patient with their partner’s brain and a stranger’s body. As for the identification question, however, significantly more participants indicated that they would save *neither* patient when the patients had lost their moral conscience, compared to when they lost their memories, *x*^2^(1) = 10.70, *p* = .001, or experienced no psychological change, *x*^2^(1) = 7.76, *p* = .005, with no difference between the control and ‘memories’ condition *x*^2^(1) = 0.33, *p* = .56 (see Table 2).

**Table 2.** Percentage of participants in Study 1 choosing to save each (or neither) patient, as a function of whether they experienced no psychological change (control condition), lost their memories, or lost their morals.

	*“The patient you choose will live; the other one will die. Who do you choose to live?”*		
	Patient with the partner’s brain, and stranger’s body	Patient with the stranger’s brain, and partner’s body	Neither
Control	81%	12%	7%
Loss of Memories	86%	8%	5%
Loss of Morals	67%	10%	23%

**Table 3.** Percentage of participants in Study 2 judging that each (or neither) patient was the ‘real’ partner, as a function of whether they experienced no psychological change (control condition), lost their memories, or lost their morals.

	*“Which of the two patients is your partner?”*		
	Patient with the partner’s brain and stranger’s body	Patient with the stranger’s brain and partner’s body	Neither
Control	65%	10%	26%
Loss of Memories	60%	9%	31%
Loss of Morals	59%	8%	33%

**Figure 2.**
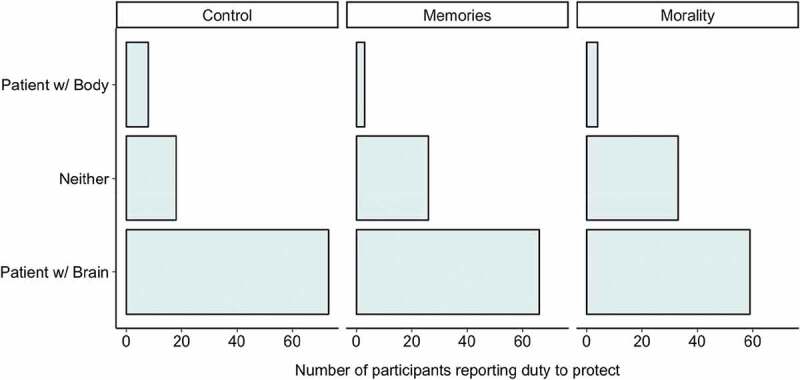
Number of participants indicating the patient they felt they had a duty to protect, broken down by whether the patient experienced no psychological change, lost their memories, or lost their moral conscience.

When asked directly about which patient they had a moral duty to protect (see Figure 2), for all conditions, most participants said that they had a duty to protect the patient with their partner’s brain, though, again, significantly more participants said they had no moral duty to protect *either* patient in the moral condition, compared to in the control condition, *x*^2^(1) = 4.41, *p*= .04. There was no difference between the ‘morality’ and ‘memories’ conditions, *x*^2^(1) = 0.83, *p*= .36, nor was there a difference between the control and ‘memories’ conditions, *x*^2^(1) = 1.45, *p*= .23.

Finally, looking at the ratings of the wrongness of sacrificing the brain-patient (see Figure 1(b)), we find a significant effect of the ‘change’ condition, *F*(2,287) = 5.22, *p*= .006, η_p_^2^ = .04, such that sacrificing the patient was seen as significantly more wrong when the patient experienced no psychological change than when they lost their moral conscience (*t*= 3.19, *p*= .005). It was marginally but non-significantly seen as more wrong to sacrifice the patient when they had lost their memories than when they had lost their moral conscience (*t*= 2.23, *p*= .068). There was no difference between the control and ‘memories’ conditions, (*t*= 0.94, *p*= .62).

#### 3.3.3. Consequences of change

**Figure 3.**
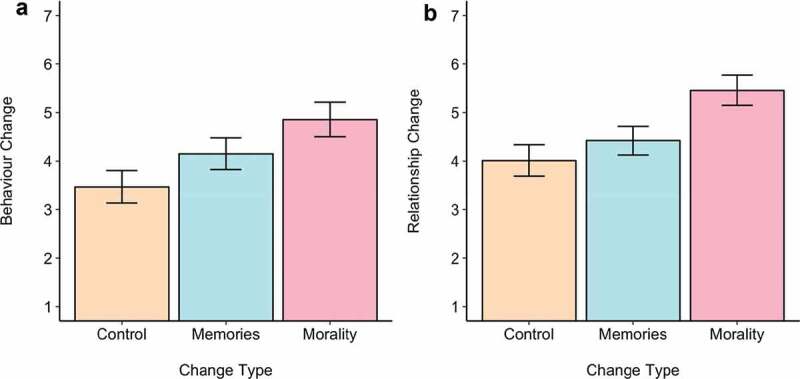
Consequences the body-switch as a function of condition (no psychological change; loss of memories; loss of moral conscience). Higher scores indicate a higher degree of change, and thus identity disruption. Error bars represent 95% confidence intervals.

Finally, all participants were asked to focus just on the patient with the partner’s brain. We looked at the perceived consequences of the psychological change relative to the perceived consequences of the physiological change: how much did participants expect that someone who lost their moral conscience or memories would behave differently, and how much would this negatively affect their relationship with this person? In line with previous work (Everett et al., Under Review), we find a significant effect of condition on expected behavior change, *F*(2,287) = 16.62, *p*< .001, η_p_^2^ = .01, with participants expecting greater behavior change after the person loses their moral conscience than when they lose their memories or experience no psychological change, all pairwise comparisons being significantly different (see Figure 3(a)). Similarly, we found a significant effect of condition on expected relationship change, *F*(2,287) = 22.67, *p*< .001, η_p_^2^ = .014, again with participants expecting that their relationship with their partner would change the most after they lost their morals than when they lost their memories or experienced no change (see Figure 3(b)). There was no significant difference in expectations of relationship change between the control and ‘memories’ condition.

### 3.4. Discussion

In Study 1, we found that, consistent with previous work, participants judged that a person’s identity would be more disrupted if they lost their moral conscience than if they lost all their memories. We also showed that these judgments might have practical consequences: a person who lost their moral conscience, more than one who lost their memories, was expected to behave more differently, and participants expected to have a worse relationship with them. Moreover, participants perceived fewer moral duties toward a loved one who lost their moral conscience than one who lost their memories. These results are consistent with – though not, of course, direct proof of – the idea that participants judged the person’s very identity to be disrupted after changes in their morality, and that they did not only judge perceptions of similarity. Even if participants were considering similarity, it seems that they were still judging it to be more disruptive for someone to lose their moral conscience than their memories, plausibly because this is a worse fate that affects more people. Given the social nature of morality, a loss of morals would be likely to affect the lives of others more negatively than a loss of memories would.

## 4. Study 2

In Study 2, we sought to replicate the results of Study 1 in a slightly different context, looking not at which patient participants thought should be sacrificed, but which patient participants thought they had a moral responsibility to care for.

### 4.1. Method

#### 4.1.1. Open science

All measures, manipulations, and exclusions, as well as all data, analysis code, and experiment materials are available for download at https://osf.io/gzywt/

#### 4.2.2. Design

292 American participants completed the survey online using Amazon Mechanical Turk and received 0.80 USD for their time. Participants were excluded from analysis if they took the survey more than once (N = 4), leaving a final sample of 288 participants (135 female, *M*_age_ = 35, *SD* = 10.80). This study was identical to Study 1 (same control, ‘memories’, and ‘morality’ conditions), except that instead of being asked which of the two patients to save with an injection, participants were asked which of the patients they should care for. More specifically, participants were shown the following text:

The doctors explain that as you are the closest person to your [partner], if you wish you can take one of the patients home and care for them until their death. This will require you taking time off work and becoming a full-time carer for them as they become progressively more and more sick. The doctors explain that they are only offering this option to you as a legal requirement, and there is no obligation to take either patient home. They are unable to find a contact for the stranger, and so the patient(s) you do not choose will remain in hospital until their death.

The dependent measures from Study 1 were adapted accordingly for this scenario (e.g., “How morally required is it to *care for* the patient with your [partner’s] brain and the stranger’s body?)

### 4.2. Results

#### 4.2.1. Identity persistence

First, we looked at identity persistence. Across all conditions, participants again adopted a psychological account of identity persistence, with most participants judging that the “real” partner was the patient with their partner’s brain and the stranger’s body, and few judging that the “real” partner was the one with the stranger’s brain but the partner’s body (< 10%; see Table 3). There was no difference in the choices between the three conditions.

Surprisingly – given the results from Study 1 and the responses to the other dependent measures – there is no difference in the ratings of psychological identity persistence between the three conditions, *F*(2,285) = 1.49, *p*= .23, η_p_^2^ = .01. Participants thought their partner’s identity was equally disrupted when they lost their moral conscience as when they lost their memories or experienced no psychological change at all.

#### 4.2.2. Moral duties

Second, we looked at participants’ perceived moral duties toward the patients. When asked who to care for, across all conditions, the majority of participants said that if forced to decide, they would care for the patient with their partner’s brain and a stranger’s body. As for the identification question, significantly more participants indicated that they would care for *neither* patient if the patient had lost their moral conscience, as compared to if the patient had experienced no psychological change, *x*^2^(1) = 6.15, *p* = .013. There is no difference, however, between the number of participants who would choose to save neither patient if they lost their memories, and the number of participants who would choose to save neither patient if they lost their morals, *x*^2^(1) = 0.62, *p*= .43. There is only a marginal difference between the ‘memories’ and control conditions, *x*^2^(1) = 2.95, *p*= .09 (see Table 4).

**Table 4.** Percentage of participants in Study 2 choosing to care for each (or neither) patient, as a function of whether they experienced no psychological change (control condition), lost their memories, or lost their morals.

	*“Which patient– if any – do you take home to care for?”*		
	Patient with the partner’s brain, and stranger’s body	Patient with the stranger’s brain, and partner’s body	Neither
Control	76%	9%	16%
Loss of Memories	61%	12%	28%
Loss of Morals	63%	5%	32%

When asked directly about which patient they had a moral duty to protect, for all conditions, most participants said that they had a duty to protect the patient with their partner’s brain, though there are no significant differences between the three conditions. Regarding the likelihood of saying that one has a moral duty toward neither patient, there was no difference between the ‘morality’ and the control conditions, *x*^2^(1) = 2.08, *p* = .15, nor between the ‘morality’ and ‘memories’ conditions, *x*^2^(1) = 0.07, *p*= .79, nor was there a difference between the ‘memories’ and control conditions, *x*^2^(1) = 1.39, *p*= .24.

Turning to the scale-ratings, there was a significant effect of the type of change in the patient on participants’ perceived duty to care for them, *F*(2,285) = 5.22, *p*= .006, η_p_^2^ = .04, with participants indicating that they had less of a moral duty to care for the patient with their partner’s brain when they had changed in terms of memories (t = 2.65, *p*= .02) or morality (t = 2.92, *p*= .01) compared to when they had experienced no psychological change, with no differences between the ‘memories’ and ‘morals’ conditions, t = 0.23, *p*= .97. Similarly, there was a significant effect of the type of change on the reported wrongness of leaving and not helping the patient with the partner’s brain, *F*(2,284) = 9.53, *p* < .001, η_p_^2^ = .06, with participants indicating that it would be less wrong to leave this patient when they had changed in terms of memories (*p*< .001) or morality (*p*= .02) than when they had experienced no psychological change, but with no difference between the ‘memories’ and ‘morality’ conditions (*p*= .26).

#### 4.2.3. Consequences of change

Finally, asking participants to focus just on the patient with the partner’s brain, we looked at the perceived consequences of the change: relative to when the patient experienced no psychological and only physiological changes, how much did participants expect that someone who loses their moral conscience or memories would behave differently, and how much would this negatively affect their relationships? In line with previous work (Everett et al., Under Review), we found a significant effect of condition on expected changes in behavior, *F*(2,285) = 15.66, *p*< .001, η_p_^2^ = .01, with participants expecting greater behavioral change when the patient lost their moral conscience than when they lost their memories or experienced no psychological change, but with no difference between the control and ‘memories’ conditions. Similarly, we found a significant effect of condition on the expected change in the relationship, *F*(2,285) = 5.71, *p* = .004, η_p_^2^ = .04, with participants expecting that their relationship with their partner would change more when they lost their moral conscience than when they experienced no change, but with no difference between the control and ‘memories’ condition, nor between the ‘memories’ and ‘morality’ conditions.

## 5. General discussion

A growing body of work has suggested that, contra the traditional philosophical view that memories are critical for identity persistence, it is instead moral values that are perceived to be central and essential for personal identity (e.g., Everett et al., Under Review; Heiphetz et al., 2017; Strohminger & Nichols, 2014). In this paper, we sought to extend this line of work, looking not just at how different changes influence abstract judgments of identity persistence, but also how these judgments have practical consequences, as well as to what extent focusing on such consequences addresses recent criticisms of the research on the moral self effect.

An initial first step in this paper was to look at direct ratings of identity persistence, testing whether personal identity is more disrupted by loss of morals than by a loss of memories (or by the lack of any psychological change). In this endeavor, we have, surprisingly, obtained mixed results. In Study 1, we replicated previous findings (e.g., Everett et al., Under Review) showing that participants did think that a person’s identity would be more disrupted if they lost their moral conscience than if they lost all their memories or experienced no psychological change. This was seen in scale ratings of identity persistence (as in previous work), but also in categorical decisions of which patient was the “real” partner after a body switch, where significantly more participants thought neither patient was the “real” partner when they lost their memories. It was surprising to us, then, that we did not replicate these results in our second pre-registered study. It is not clear to us why this is so, given that the dependent measures and written instructions are almost identical between the two studies, we used the same analyses in both studies, and this general pattern of results has been widely replicated in previous research across a variety of contexts.

Regardless, another central aim in this paper was to look at the practical consequences of identity-change judgments and the extent to which these judgments influence (a) participants’ perceptions of moral duties toward their partner, (b) perceived relationship quality, and (c) expectations of future behavior change. Again, the results from Study 1 support our predictions: participants perceived their moral duties toward their partner to be reduced when the partner had lost their moral conscience, they expected their partner to behave more differently when they lost their morals than when they lost memories or experienced no psychological change, and they expected their relationship with their partner to change more if the partner lost their morals than if they lost their memories.

Results from Study 2 did not show the same pattern – likely in large part because we failed to replicate the basic finding that people view a loss of morals as more disruptive for identity than a loss of memories. Participants were quite consistent in thinking that their moral duties toward the patient were reduced when the person changed, but there were few differences in the perceived moral duties toward a patient who lost their memories versus one who lost their morals. Despite the mixed nature of this particular result, our findings still advance the recent debate about qualitative versus quantitative identity, which we discuss in more detail below.

One final noteworthy feature of our work is that we did partially replicate previous results using a different paradigm than is usually employed. Typically, this work has used a within-subjects paradigm in which participants are presented with a series of different types of changes and asked to rate for each change how much a person would be a different person after changing in that way, assuming that everything else about them remained the same (though, for an exception, see Study 1 of Strohminger & Nichols, 2015). One might argue that presenting these changes in this manner is not strong enough to bring about an impression of real identity change. That is, losing a single memory might not disrupt identity, but losing a whole series of connected memories – as in complete amnesia, like in our experiments – might indeed make someone a different person. That we observe the moral self effect (at least in Study 1) when using the alternative within-subjects paradigm provides further evidence that the effect is robust and not simply an artifact of the particular experimental paradigm typically used.

## 6. Similarity or identity?

Starmans and Bloom (2018) have claimed that previous empirical work on the moral self effect is about similarity and not personal identity. We wanted to see if we could address this challenge empirically by leveraging the idea of special obligations, or the duties one has to someone simply because of who they are. Ethically, we might think that I have certain obligations to my mother which I don’t have to a stranger, and these special obligations toward my mother do not change even if she were to suffer from a severe, debilitating stroke that changed her personality. My obligations and duties to her are the same because she herself *is* the same person, however dissimilar she is now to how she was in her prime. In this way, if participants are thinking at least to some extent about identity and not just similarity, we should expect that judgments about moral duties should partially track judgments of change: when participants judge that someone is more of a different person when they lose their morals than when they lose their memories, they should also be more likely to say that their moral duties toward that person are more reduced when they lose their morals than when they lose their memories. If, in contrast, Starmans and Bloom are correct to say that in these kinds of experiments, participants are just thinking about similarity and not identity, then participants should rate someone who loses their morals as more different without perceiving any changes in their own moral duties toward that person.

In Study 1, we find results that are consistent with the idea that participants think about both identity and similarity.^2^ When asked who to save, the patient with the stranger’s brain and partner’s body or the one with the partner’s body and the stranger’s brain, participants who were told that the patient had completely lost their morals were more likely to say that they would save neither patient, compared to participants who were told that the patients had lost their memories or experienced no psychological change. Similarly, sacrificing the patient with their partner’s brain and the stranger’s body was seen as significantly more wrong when the patient experienced no psychological change than when they lost their moral conscience, and it was seen as marginally but non-significantly more wrong to sacrifice the patient when they had lost their memories than when they had lost their moral conscience.

When asked to make a categorical decision about which patient they had a moral duty to protect, significantly more participants said they had no moral duties to protect either patient in the ‘moral’ condition, as compared to the control condition, though there was no difference between the ‘morality’ and ‘memories’ condition. Across all measures, participants felt their moral duties to be largely preserved toward someone who experienced no psychological change, yet more disrupted when the person changed, though the difference between memories and morals was not always significant.

Overall, these empirical results show a clear link between perceived identity change and perceived moral duties, suggesting that it is not possible to entirely dismiss previous demonstrations of the moral self effect as being only about similarity. This is to be expected, given the low likelihood that lay-people are spontaneously and intuitively activating subtle philosophical distinctions between, for example, quantitative and qualitative identity. Instead, we think, participants are likely to think about the self in terms of both identity and similarity, flexibly activating and focusing on either qualitative or quantitative identity in different contexts depending on the task at hand.

We also think, however, that Starmans and Bloom’s challenge can be resisted on conceptual grounds. In the first place, we are not sure that it is entirely possible to separate qualitative identity (similarity) from quantitative identity (personal). Indeed, part of the reason that we have statutory limitations on prosecuting certain crimes seems to be that even if the transgressor shares the same numerical identity, their qualitative similarity has dropped enough to sever the link with moral responsibility (Mott, 2018; Tobia, 2016).

Moreover, we are not sure that it is entirely possible to separate qualitative (similarity) from quantitative (personal) identity within prominent philosophical treatments, such as the psychological reductionist account of personal identity proposed by, for example, Parfit (1984). These accounts suggest that what matters is not the strict identity relation of memories between persons (e.g., where Person is at Time-1 is *either* identical to where Person is at Time-2, *or* they are different persons) but the degree of psychological connectedness. According to Parfit (1984), such psychological connectedness does include memories, but it also includes psychological dispositions, attitudes, personality, preferences, and so on. This conception has elements of qualitative identity (a greater degree of psychological connectedness means that someone is more similar), but also quantitative identity, as it is the degree of psychological connectedness that allows one to identify a person at different times as the same person. Both of these points suggest that a strict division between qualitative identity (similarity) and quantitative identity (personal) is likely untenable. Starmans and Bloom may be right in saying that previous findings are partly about similarity, but this does not mean that they do not tell as anything about ordinary peoples’ intuitions regarding personal identity, at least on a psychological continuity account.

## 7. Limitations and future directions

We have argued that previous findings cannot be explained solely in terms of similarity, but also that participants’ responses are reflecting – at least partially – their intuitions about personal identity. In the present design, it is impossible to entirely tease apart similarity from personal identity (to see to what extent this is possible at all, consult the above discussion), and it would be interesting for future work to explore further the relative influence of moral changes on similarity and personal identity separately.

By focusing on perceived moral duties toward targets undergoing changes, our paper also raises a range of new questions about how different kinds of identity changes influence perceptions of the target’s own moral responsibility and accountability. Imagine, for example, that prior to the brain transplant, the stranger had committed a crime. Presumably, participants would hold the patient with the stranger’s brain to be morally accountable for the crime; however, would this effect be weaker if the patient loses their memories or moral conscience in the process of the transplant? Future work should explore these and related questions, which bring the practical import of judgments of identity change to the fore.

Returning to more everyday matters, our results might explain why people regard patients with Alzheimer’s disease and other dementias as being the same person, even when they have lost substantial memories. Provided their moral character remains intact, they are regarded as being the same. Indeed, it is when a person’s *moral behavior* changes with dementia that they are typically said to be “not themselves” – not when they lose their memory. Presumably, this is why participants judged it, marginally, to be more wrong to sacrifice the patient when they had lost their memories than when they had lost their moral conscience. It seems that a loss of one’s moral conscience is worse than a loss of memories: worse for the one with the loss because others will treat them differently, and worse for others because people without morality do not typically act in ways that benefit or respect others.

Finally, it is important to reiterate that our work here seeks to shed light on ordinary people’s intuitions about personal identity and moral duties and not to draw metaphysical conclusions about the nature of personal identity per se. We show that ordinary people think that morality is important for psychological continuity and that this judgment is related to subsequent perceptions of moral duties. It is possible that people are mistaken about the nature of personal identity or their moral duties, but that is a debate for another paper.

## Notes

1. For completeness, we also asked participants to indicate how much the patient with the partner’s body and the stranger’s brain was the real partner, but we do not report this in the main manuscript, given that participants overwhelmingly adopted a psychological view of identity persistence. Similarly, while we also asked participants to indicate how wrong it would be to sacrifice the patient with the partner’s body and the stranger’s brain, in the interests of space and clarity we focus on judgments of sacrificing the brain-patient. Results for these additional questions are reported in the supplementary results.

2. In Study 2, participants did not show the moral self effect; thus, it is impossible for us to make conclusions about whether this effect is due to similarity or identity – there is no observed effect for us to tease apart.
